# Renal function and risk factors for renal disease for patients receiving HIV pre-exposure prophylaxis at an inner metropolitan health service

**DOI:** 10.1371/journal.pone.0210106

**Published:** 2019-01-17

**Authors:** Douglas Drak, Hamish Barratt, David J. Templeton, Catherine C. O’Connor, David M. Gracey

**Affiliations:** 1 Sydney Medical School, University of Sydney, Sydney, NSW, Australia; 2 Central Clinical School, University of Sydney, Sydney, NSW, Australia; 3 RPA Sexual Health, Sydney Local Health District, Sydney, NSW, Australia; 4 Kirby Institute, University of New South Wales, Sydney, NSW, Australia; 5 Renal Unit, Royal Prince Alfred Hospital, Sydney Local Health District, Sydney, NSW, Australia; Temple University, UNITED STATES

## Abstract

**Background:**

Pre-exposure prophylaxis (PrEP) with tenofovir disoproxil fumarate/emtricitabine (TDF/FTC) significantly reduces the risk of HIV acquisition. TDF is a known nephrotoxin however, renal dysfunction from TDF is mostly reversible following discontinuation.

**Aims:**

To describe the renal function, risk factors for renal disease and associated clinical testing practices in a cohort of PrEP patients.

**Methods:**

A retrospective review was conducted of all PrEP patients commenced on TDF/FTC at an inner metropolitan sexual health clinic in Sydney, Australia between April 2016 and July 2017, with follow-up data obtained at 3-monthly intervals until 18 months.

**Results:**

525 patients met inclusion criteria. Patients were almost exclusively male and median age was 34 years (IQR: 28 to 42). At baseline, 1.5% had an estimated glomerular filtration rate (eGFR) <70 mL/min/1.73m^2^. A small significant drop in eGFR of -2.5 mL/min/1.73m^2^ (p<0.05) occurred between PrEP commencement and the first follow-up period, followed by a progressive decline in eGFR of -0.38 mL/min/1.73m^2^ per month (95%CI: -0.57 to -0.20; p<0.001). Renal impairment (eGFR <70 mL/min/1.73m^2^) occurred in 6.5% of patients and persisted across consecutive follow-up periods in five (1.0%) patients. Patients aged ≥40 years had a greater risk of renal impairment than younger patients (HR 3.9, 95%CI: 1.8 to 8.4; p<0.001), despite similar rates of eGFR decline (p = 0.19). PrEP was discontinued in two patients (0.4%) due to renal function concerns.

**Conclusion:**

PrEP use led to an initial drop in eGFR and a more gradual progressive decline subsequently, but significant renal impairment remained uncommon up to 18 months of follow-up.

## Introduction

Fixed-dose combination tenofovir disoproxil fumarate (TDF) and emtricitabine (FTC) for pre-exposure prophylaxis (PrEP) has been found to be highly efficacious in HIV prevention, reducing the risk of HIV acquisition by 86% [1, 2]. Many countries have since approved TDF/FTC for this indication [3], including Australia in May 2016.

TDF is associated with a number of adverse events; however, nephrotoxicity is a particular concern. Nephrotoxicity typically manifests first as proteinuria, followed by a progressive decline in estimated glomerular filtration rate (eGFR) [4]. In rare instances, severe dysfunction results in Fanconi Syndrome [5]. Renal dysfunction from TDF, detected within the first few months, is at least partially reversible in the majority of patients upon discontinuation of the drug [6]. As such, routine monitoring of renal function is critical in patients receiving TDF.

Presently, a large open-label clinical trial, the Expanded PrEP Implementation in Communities in New South Wales (EPIC-NSW; NCT02870790), is underway. This trial has involved a rapid rollout of PrEP to those at high-risk of HIV infection and was designed to approximate “real-world” use of PrEP [7]. A recent audit, of one of the main clinics involved, showed the clinic’s HIV-positive population had a high prevalence of renal risk factors [8], many of which are likely shared by patients at high-risk for HIV infection.

Here we describe the renal function, risk factors for renal disease and the testing regimens in place for a population at high-risk for HIV infection and receiving PrEP at a metropolitan sexual health service, based in Sydney’s inner west.

## Methods

This study was conducted in accordance with the Declaration of Helsinki and applicable local and national guidelines. Ethics approval for this project was obtained from the RPAH Zone Human Research Ethics Committee of the Sydney Local Health District. This included a waiver of consent, permitting access to identified health data.

A retrospective case note review was conducted of all patients attending RPA Sexual Health (RPASH) and enrolled in the EPIC-NSW study, commenced on PrEP between April 2016 and July 2017. Patients who had been on TDF-containing post-exposure prophylaxis or had received PrEP via private prescription for one month or less immediately prior to enrolling in EPIC-NSW were also eligible for inclusion. Follow-up data were collected up to 18 months after commencement.

In the EPIC-NSW study, from which our patient population was drawn, participants were included if they met criteria for high-risk behaviour for HIV infection. Participants were excluded from the EPIC-NSW study if they tested HIV positive, had symptoms consistent with HIV infection seroconversion illness, or an eGFR of less than 60ml/min/1.73m^2^. The detailed methodology of the EPIC-NSW study has been published elsewhere [7].

Follow-up review of renal function occurred every three months, at which time PrEP was dispensed. Each missed follow-up was therefore taken to equate to 3 months of missed medication. Where multiple tests were performed within a three-month follow-up period, the mean of the combined test results was calculated to provide a single value for that testing interval.

Renal function data were collected at baseline, prior to PrEP commencement, and at follow-up visits. Risk factors for renal disease were assessed at baseline.

### Renal function

eGFR was calculated using the CDK-EPI equation, which has been shown to provide better estimates of eGFR in patients with normal renal function [9]. Temporal trends in renal function indicators were analysed chronologically from study enrolment (unadjusted), cumulative duration of PrEP exposure (adjusted) and by censoring patients at first missed follow-up to approximate continuous PrEP exposure. All analyses refer to unadjusted data unless otherwise specified.

Three different thresholds were used to define renal dysfunction: eGFR <60 mL/min/1.73m^2^—cut-off for CKD), eGFR <70—used in previous studies to define renal impairment [4] and a decline in eGFR >25% from baseline—a marker of clinically significant kidney injury [10]. Proteinuria was defined as a urinary protein to creatinine ratio (uPCR) of >20mg/mmol [6] and hypophosphatemia as serum phosphate <0.8 mmol/L.

### Testing protocols

To evaluate testing practices, criteria that would warrant further review of patients were applied retrospectively. Patients were said to require repeat testing, within two weeks, if: a >25% decline in eGFR from baseline, an eGFR newly <60 mL/min/1.73m^2^ and/or a significant proteinuria (uPCR >20 mg/mmol) was noted. Criteria for nephrologist review were: a >30% decline in eGFR and/or eGFR newly <60 mL/min/1.73m^2^ persisting over a period of at least three months.

### Statistical analysis

Statistical analysis was performed using SPSS version 20 (IBM, USA). Changes in renal function indices from baseline and between testing intervals were assessed using a two-tailed t-test. Simple linear regression was applied to determine the relationship between eGFR and uPCR. Lastly, potential predictors of renal function decline (eGFR <70 mL/min/1.73m^2^ or eGFR decline >25% from baseline), were assessed by log-rank test. For this test, missing data were imputed by calculating the midpoint of adjacent data points and patients were censored at the last 3-monthly appointment within the 18-month study period where data were available. eGFR values are expressed as mean ± SEM. For all analyses, a p-value <0.05 was considered statistically significant.

## Results

A total of 525 patients met the criteria for inclusion in this study. The patient cohort is summarised in Table 1. It was almost exclusively male, but included three transgender individuals. Median patient age was 34 years (IQR: 28 to 42). The total follow-up time was 562 person-years, with median follow-up of 15 months (IQR: 9 to 18) per patient. Approximately one third of patients were currently using recreational drugs, of which 1 in 6 were injecting drug users. Ninety-nine patients (19%, 95%CI: 16 to 22) had a baseline eGFR under 90, but greater than 60 mL/min/1.73m^2^. Patients were excluded from the EPIC-NSW study, if their baseline eGFR was <60 mL/min/1.73m^2^.

**Table 1:** Patient characteristics (n = 525).

Characteristic	Number (%)
Sex	
Male	522 (99.4)
Transgender	3 (0.6)
Age (years)	
<30	163 (31.0)
30–39	189 (36.0)
40–49	121 (23.0)
≥50	52 (9.9)
Baseline eGFR (mL/min/1.73m^2^)	
>90	426 (81.1)
80–90	65 (12.4)
70–80	26 (5.0)
<70	8 (1.5)
Medically Advised PrEP Discontinuations	4 (0.8)
Recreational Drug Use	
Current	182 (34.7)
Former	60 (11.4)
Never	283 (53.9)
Intravenous Drug Use	
Current	28 (5.3)
Former	30 (5.7)
Never	467 (90.0)

PrEP discontinuation was defined as discontinuation due to no longer meeting the medical eligibility for the EPIC-NSW study and/or having been medically advised to discontinue PrEP; GFR–estimated glomerular filtration rate, PrEP–pre-exposure prophylaxis

### Therapy tolerability, discontinuations and interruptions

Adverse events prompted two patients (0.4% of total; 95%CI: 0.0 to 0.9) to self-cease their medication. A further 13 (2.5% of total, 95%CI: 1.1 to 3.8) permanent PrEP discontinuations were recorded. Nine patients were no longer at sufficiently high-risk of HIV infection to continue in the EPIC-NSW study and four patients were medically advised to stop PrEP due to low eGFR (two patients), deranged hepatic function (one patient) and newly diagnosed malignancy (1 patient). Fourteen patients had their care transferred to other clinics and 108 patients (21%, 95%CI: 17 to 24) had no follow-up appointments for at least six months This latter group may have discontinued PrEP and thus were censored from the analysis at that point.

Missed appointments and planned interruptions in PrEP, were common. Nearly one third (n = 160, 30%, 95%CI: 27 to 34) of patients did not attend least one follow-up appointment, with 1 in 20 (n = 19, 3.6%, 95%CI: 2.0 to 5.2) missing two or more appointments. Therapy interruptions were more common in patients aged <40 years than patients ≥40 (34% vs 23%, p<0.001).

### Renal function

**Fig 1:**
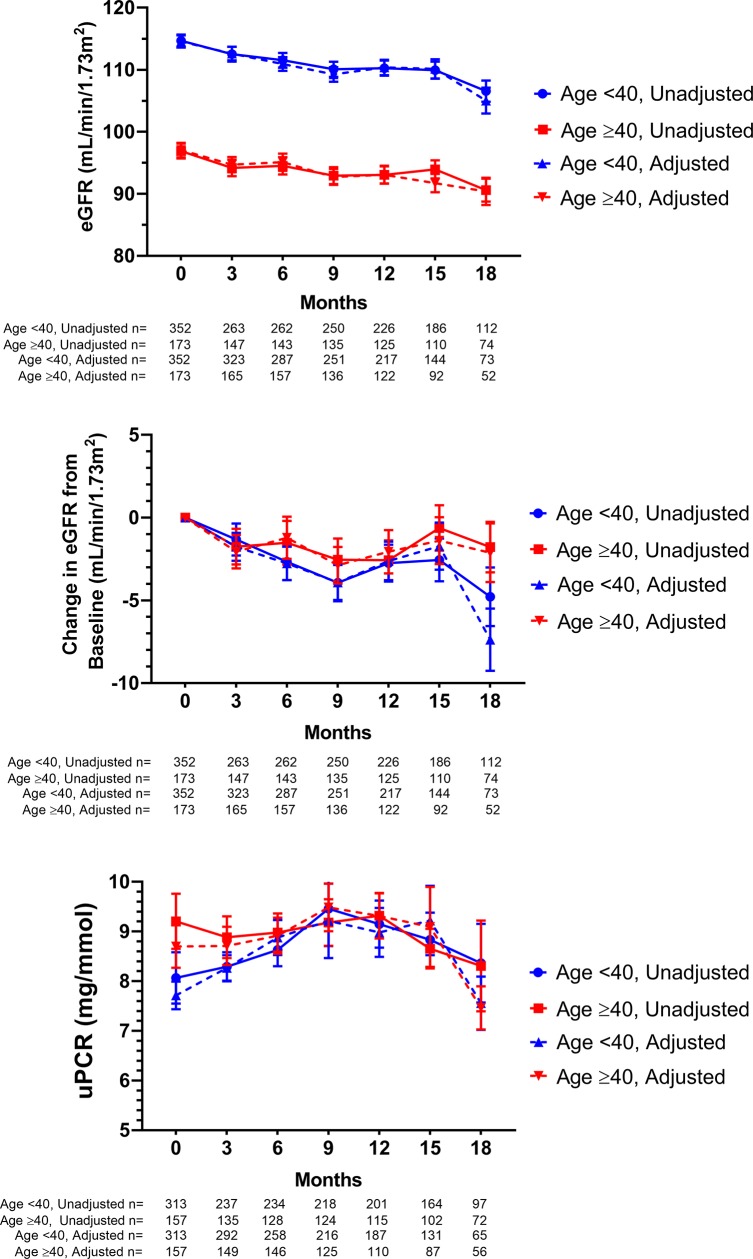
Change in renal function indices over time by age group. Time shown is months since enrolment in the EPIC-NSW trial (unadjusted) or cumulative months PrEP exposure (adjusted). The latter accounted for any temporary interruptions of medication. All graphs show mean ± SEM. eGFR—estimated glomerular filtration rate, uPCR—urinary protein to creatinine ratio.

**Fig 2:**
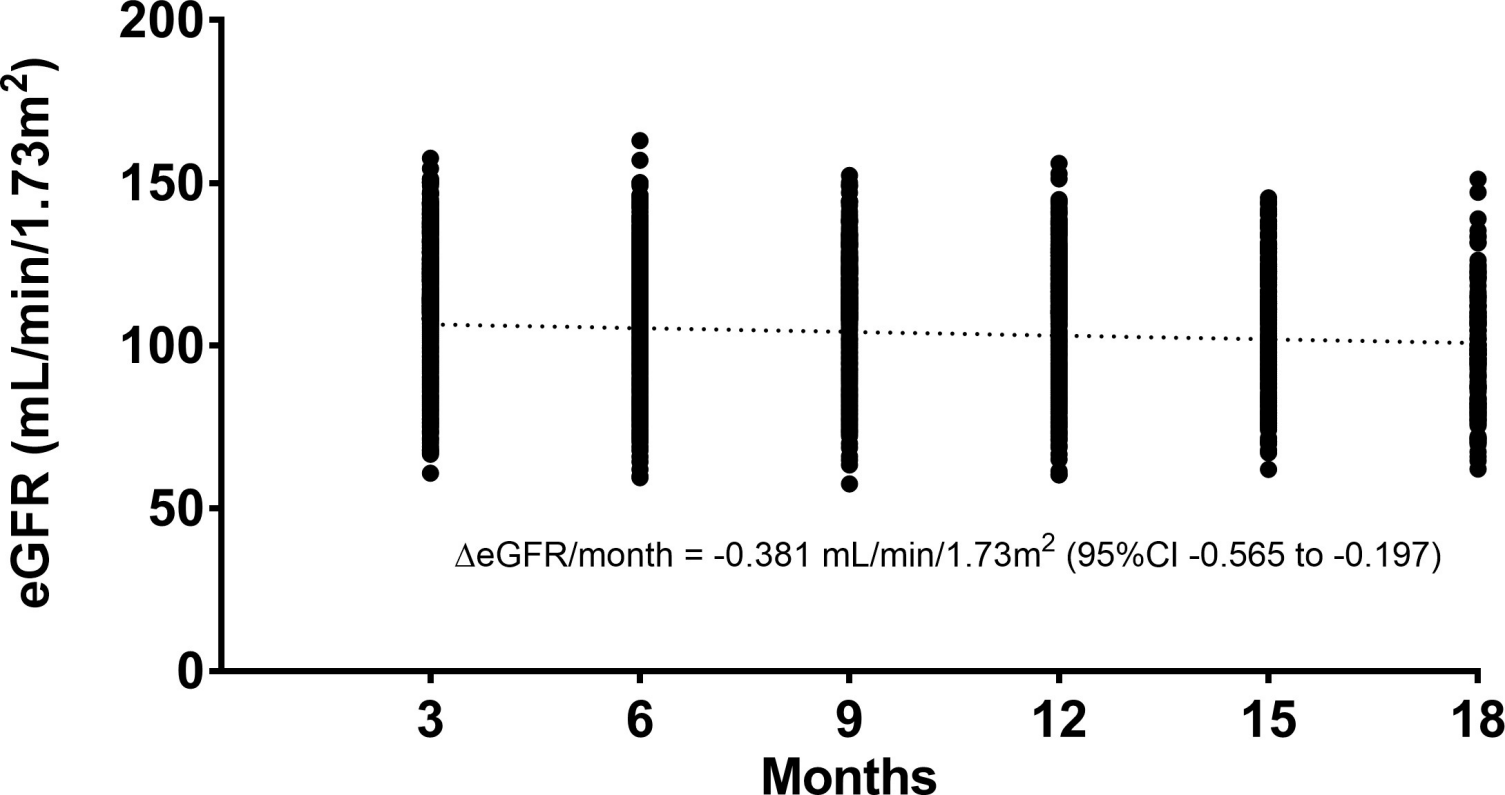
Estimated glomerular filtration rate (eGFR) at each follow-up period by individual patient, measured from time of enrolment. Trend line derived by linear regression (p<0.001).

Patients had reduced eGFR compared to baseline throughout the duration of the study (Fig 1). This was statistically significant at all time points (p<0.05), and borderline significant at 15 months (p = 0.056). In contrast, there were no significant changes in uPCR from baseline. The decline in eGFR, averaged across all follow-up time points was -2.5 ± 0.4 mL/min/1.73m^2^. The majority of this decline occurred between PrEP commencement and the first follow-up at 3 months. However, there was also a more gradual reduction in eGFR across the subsequent follow-up periods (Fig 2) of -0.38 mL/min/1.73m^2^ per month (95%CI: -0.56 to -0.20; p<0.001).

Patients aged <40 years had a significantly higher baseline eGFR than patients ≥40 (115 ± 1 vs 97 ± 1 mL/min/1.73m^2^) and across all follow-up time points (p<0.001 for all). Nonetheless, the decline in eGFR was similar for both age groups (-2.8 ± 0.5 vs -1.8 ± 0.5 mL/min/1.73m^2^, p = 0.26).

Trends for eGFR and uPCR were similar whether measured chronologically from study commencement or adjusted for duration of PrEP use (Fig 1). Linear regression showed significant correlation between eGFR and uPCR at baseline only (p<0.001). Adjusted for duration of PrEP exposure, uPCR and eGFR were also significantly associated at 12 months (p<0.01). Censoring patients at their first missed 3-monthly appointment however, approximating continuous PrEP usage, led to significant associations at all timepoints (p<0.05 for all).

Individual test results showing significant renal impairment was common, with nearly one- fifth of patients (n = 98, 17%, 95%CI: 15 to 22) meeting one of the three criteria for an abnormal eGFR. When repeat tests within follow-up periods were averaged, three patients (0.6%, 95%CI: 0.0 to 1.2) had eGFR values <60 mL/min/1.73m^2^, 34 (6.5%, 95%CI: 4.4 to 8.6) had values <70 mL/min/1.73m^2^ and eGFR declines >25% from baseline were noted in 72 patients (14%, 95%CI 11 to 17). These persisted for over 3 months in 0, 5 and 10 patients respectively. Proteinuria was identified in 64 patients (12.2%, 95%CI: 9.4 to 15.0) and was not persistent in any case. Testing of serum phosphate was infrequent, but 6/61 (9.8%, 95%CI: 2.4 to 17.3) tested patients had at least one instance of hypophosphatemia (<0.8 mmol/L), persisting in a single patient.

### Renal risk factors

Age ≥40 years was associated with an increased risk of eGFR <70 mL/min/1.73m^2^ (HR 3.94, 95%CI: 1.85 to 8.40), but not an eGFR decline >25% from baseline (HR 0.82, 95%CI: 0.50 to 1.32). Among those with baseline eGFR <90 mL/min/1.73m^2^, there was a substantially reduced risk of a >25% decline in eGFR (HR 0.16, 95%CI: 0.09 to 0.28; p<0.001), but no significant difference in the risk of developing an eGFR <70 mL/min/1.73m^2^ (HR 0.46, 95%CI: 0.19 to 1.14) during follow-up. Neither proteinuria (p = 0.61) nor current recreational drug use (p = 0.34) were associated with increased risk of renal impairment using any of the cut-offs for impaired eGFR.

Other traditional risk factors for renal disease, including smoking, blood pressure and diabetic status were rarely recorded. Ten patients were receiving antihypertensive therapy at baseline, six patients were receiving a statin and three were receiving an oral glucose lowering agent.

### Testing practices and specialist review

Repeat testing was common, with 78 (15%, 95%CI: 12 to 18) of patients having attended two or more visits within any 3-month follow-up period. Sixteen patients were referred to a nephrologist for review and further investigation; 11 were referred for declines in eGFR alone and 3 for decline in eGFR and proteinuria.

Sixty-three (12.0%, 95%CI: 9.2 to 14.8) patients met criteria for, but did not receive, retesting due to uPCR >20 mg/mmol (53 patients) a drop in eGFR from baseline of >25% and/or eGFR newly <60 mL/min/1.73m^2^ (6 patients), and eGFR decline plus proteinuria (4 patients). In all instances, these results had normalised by the next 3-monthly visit, thus specialist nephrologist review was not indicated.

## Discussion

TDF/FTC containing PrEP was associated with a small persistent decrease in eGFR and cessation of PrEP due to severe renal impairment was rare. Thus, our findings support those of previous clinical trials and observational studies.

The magnitude of the eGFR decline following TDF commencement has been remarkably consistent across previous studies despite substantial variations in study populations including sex, ethnicity and background renal impairment. The 2.5 mL/min/1.73m^2^ decline in our study was approximately midway within previously described ranges [4, 11, 12].

Following the initial decline in eGFR with PrEP commencement, a more gradual decline in eGFR was observed across subsequent follow-up visits of -0.38 mL/min/1.73m^2^ per month. This same trend was also evident in the iPrEx open-label extension (OLE) study, with a similar rate of progressive decline in creatinine clearance between 3 and 12 months. This decline began to reverse subsequently however, despite continued PrEP use [4]. Other studies have reported different patterns of renal function decline. In the Partners PrEP Study, a large randomised placebo-controlled trial, a progressive eGFR the decline only started after 18 months of exposure, but disappeared when data collected after discontinuation of its placebo arm were included [11]. Neither the iPrEx main study nor the recent US PrEP Demonstration study found any evidence of progressive eGFR decline [5, 12]. These apparently differing observations may have resulted from variations around a depressed, but stable, mean eGFR. Alternatively, the absence of a progressive decline some studies may reflect insufficient power to reliably detect a relatively small change in renal function in the context of comparably large interpatient variability.

The potential for long-term eGFR decline in PrEP patients is similarly unclear, due to a paucity of long-term data, with the longest study reporting outcomes up to only three years [11]. This is an important focus for future study, as longer-term data from HIV-positive patients have indeed noted a progressive decline in eGFR due to TDF [13, 14]. Another study, notably where all patients had normal renal function at baseline (eGFR >90 mL/min/1.73m^2^), reported the risk of development of chronic kidney disease as 14% per year of TDF exposure [15].

Renal impairment (eGFR <70 mL/min/1.73m^2^) [4], was rare in our study, occurring in 2% of patients. This low incidence in over 500 patient-years of follow-up is a reassuring finding. Other observational PrEP cohorts have reported frequencies of renal impairment of 1, 3 and 15% [4, 12, 16]. The upper end of this range is likely explained by nearly one third of that study population having pre-existing renal dysfunction (eGFR <90 mL/min/1.73m^2^), limiting its applicability to our cohort.

Despite the nephrotoxic effects of recreational drug use [17] no association was found in this study between drug use and renal dysfunction. While analyses of other PrEP cohorts [12, 16] have simialrly found no association with renal impairment, this may be a consequence of substantial heterogenetiy in drug use patterns and lack of long-term follow-up data.

The infrequent recording of our patients’ traditional risk factors for renal disease, including hypertension, smoking status and diabetes, meant we were unable to determine the impact of these parameters on renal function. Other studies have found that these risk factors do not predict greater decline in renal function in patients exposed to TDF; the key risk factors being older age and pre-existing renal dysfunction [4, 12, 16]. This apparent non-association of many traditional renal risk factors may, however, be simply a consequence of insidious contributions to renal function decline and the relatively short duration of reported PrEP studies.

In our study, patients aged ≥40 were at increased risk of developing an eGFR <70 mL/min/1.73m^2^, but were not at increased risk of a >25% decline in eGFR from baseline. The former is readily explained by the lower baseline renal function in older patients. The latter finding may have been artefactual and related to this study’s counterintuitive finding that a baseline eGFR <90 mL/min/1.73m^2^ was associated with a substantially lower risk (HR 0.16) of an eGFR decline >25%.

As eGFR values >90 mL/min/1.73m^2^ were reported to clinicians as “>90”, this would have served to conceal the magnitude of any eGFR declines, some of which should have prompted retesting. In patients with baseline eGFR values below this threshold, the magnitude of such drops were evident and retesting, when indicated, was routine. This explanation assumes that the majority of the eGFR declines were spurious or transient and thus would not recur on retesting, which is supported by 14% of patients in this study having a substantive eGFR decline (>25% of baseline), but the decline was only persistent among 2% of individuals.

Current state PrEP guidelines recommend that eGFR be tested 3-monthly [18], whereas the Australasian Society for HIV, Viral Hepatitis and Sexual Health Medicine has suggested 6-monthly testing is sufficient in patients aged 25–40 years, with a baseline eGFR >90 mL/min/1.73m^2^, who are at lower risk of renal dysfunction [19]. Our findings generally support this recommendation, despite the potential for missed substantial eGFR declines in these “lower risk” patients. Such events are likely to be of limited clinical consequence, given the reversibility of eGFR declines demonstrated in the Partners PrEP study. There, patients almost exclusively had a baseline eGFR >90 mL/min/1.73m^2^ and similar rates of persistent substantive eGFR decline (>25%) with PrEP to our study [11]. By 8 weeks following PrEP discontinuation however, no patients had an eGFR <75% of baseline and nearly all returned to an eGFR >90 mL/min/1.73m^2^ [6].

The design of the EPIC-NSW study, from which our patient population was drawn, was permissive of missed follow-up appointments and interruptions of PrEP [7] to allow for better modelling of “real-world” use of PrEP by patients. Despite the potential for variability, trends in eGFR and uPCR were similar, whether measured chronologically from study enrolment or adjusted for duration of PrEP exposure. This may be partly explained by the variable adherence to daily PrEP. Although not measured in this study, TDF concentrations in hair samples of patients on daily PrEP were at levels equivalent to ≥4 tablets/week in only 49% of patients in the iPrEx open-label extension [4] and 82% in the US PrEP Demonstration Project [12]. Intermittent PrEP usage may explain some of the self-limiting episodes of renal dysfunction observed in our study.

Although uPCR was significantly elevated from baseline for the majority of the study period, a consistent association between eGFR and uPCR was only apparent when patients were censored at the first missed follow-up appointment to approximate continuous PrEP use. Even with such adjustment, uPCR was not able to predict eGFR at future time points. The Partners PrEP Study similarly found that TDF exposure increased the rate of proteinuria, but that proteinuria did not increase the risk of a clinically significant decrease in eGFR [20]. Together, these findings suggest that uPCR is unlikely to be of use in regular monitoring of patients receiving TDF containing PrEP.

Approximately one fifth of patients were considered lost to follow-up, having missed two or more consecutive appointments, and may have permanently discontinued PrEP. While this is a sizable proportion of patients, there is nothing to suggest that renal function in this subgroup would be substantially different from the four fifths of patients who remained in the study or discontinued PrEP for documented reasons. Similarly, tolerability of PrEP this subgroup is unlikely to have been particularly poor, based on findings from earlier clinical trials [1, 2, 21]. Lifestyle factors, being common reasons for interrupting PrEP in this study, likely also explain the majority of patients lost to follow-up.

## Conclusion

Commencement of TDF/FTC containing PrEP led to a small rapid decline in eGFR initially, followed by a more gradual decline for the duration of the study period. PrEP was rarely associated with substantial renal impairment. This was more common in patients over 40 years of age, despite similar rates of eGFR decline in younger and older patients, due to lower baseline renal function in the latter. Retesting of patients with abnormal eGFR and uPCR results and referral for specialist nephrology review was generally appropriate.

## Abbreviations

eGFR: estimated glomerular filtration rate
FTC: emtricitabine
PrEP: pre-exposure prophylaxis
TDF: tenofovir disoproxil fumarate
uPCR: urinary protein to creatinine ratio
